# Supplementary figures and images for: Balancing complexity, performance and plausibility to meta learn plasticity rules in recurrent spiking networks
Source: PLoS Comput Biol. 2025 Apr 24;21(4):e1012910. doi: 10.1371/journal.pcbi.1012910 (PMC12021293; doi:10.1371/journal.pcbi.1012910)

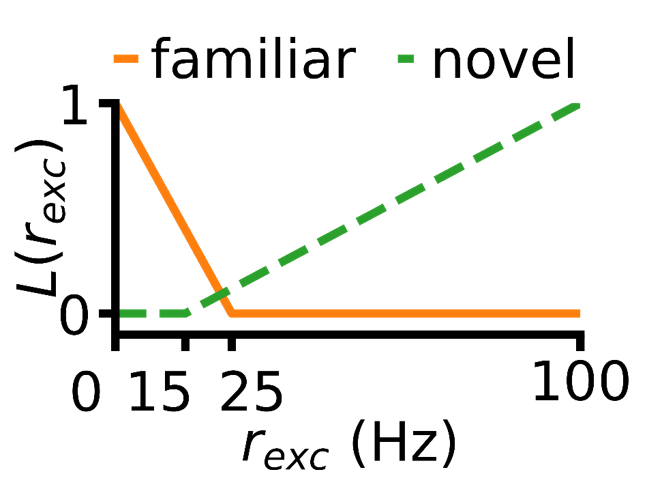

Supplement: S1 Fig — Visualization of the loss function used for the familiarity task (PNG) [file pcbi.1012910.s001.png]

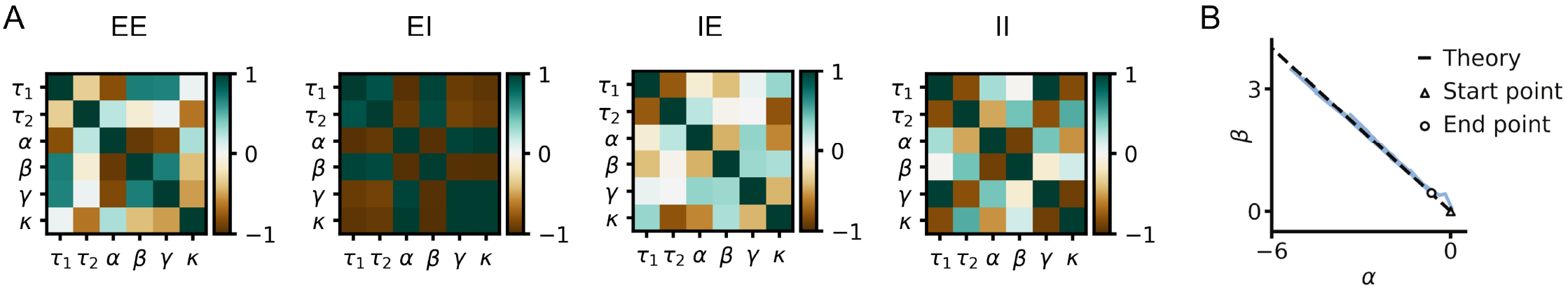

Supplement: S2 Fig — (A) Covariance matrices at the last meta-iterations for the optimizations shown in Fig 2 and 3. (B) Optimization from Fig 2C, evolution of two plasticity parameters during the optimization trajectory. Dotted line is the mean-field theoretical prediction with the non-Hebbian terms only (same analysis as in [31]). This suggests that the task is being solved mainly via the two non-Hebbian parameters, an interpretation in line with the covariance matrix visualization (PNG) [file pcbi.1012910.s002.png]

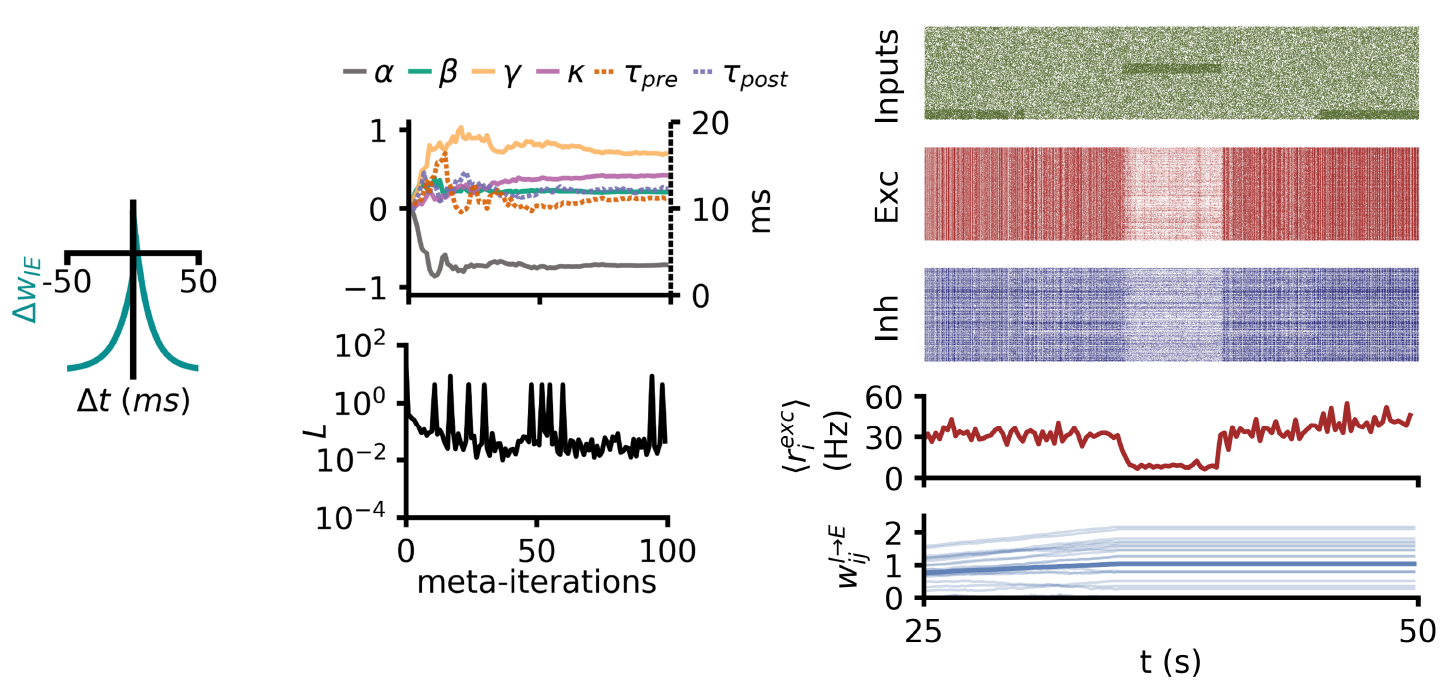

Supplement: S3 Fig — More details about the optimization shown in Fig 8A (PNG) [file pcbi.1012910.s003.png]

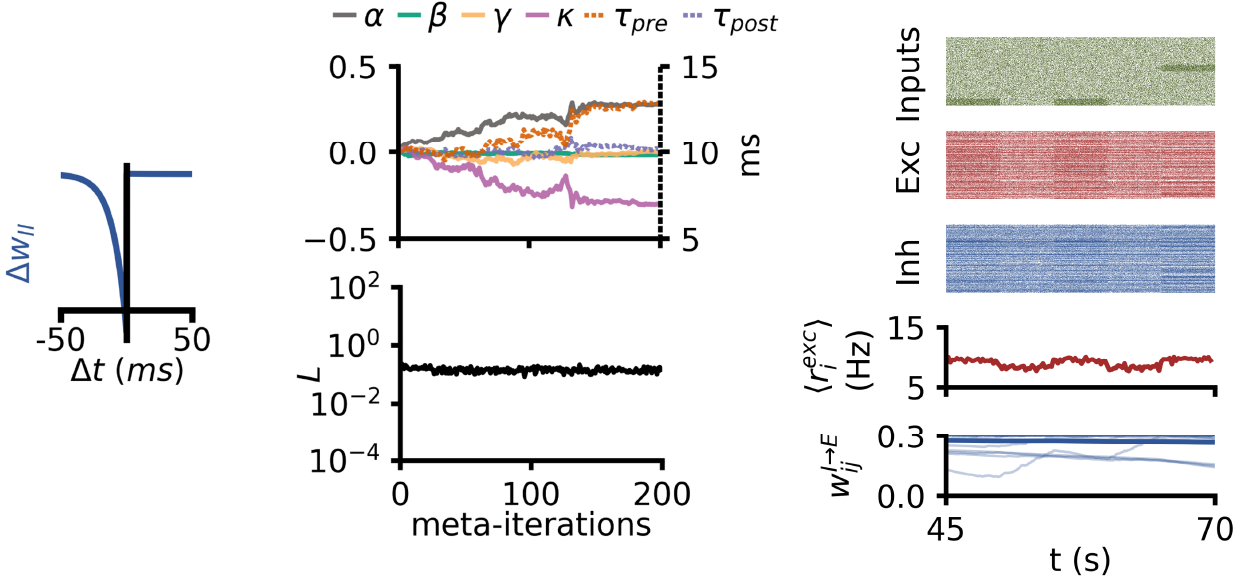

Supplement: S4 Fig — More details about the optimization shown in Fig 8B (PNG) [file pcbi.1012910.s004.png]

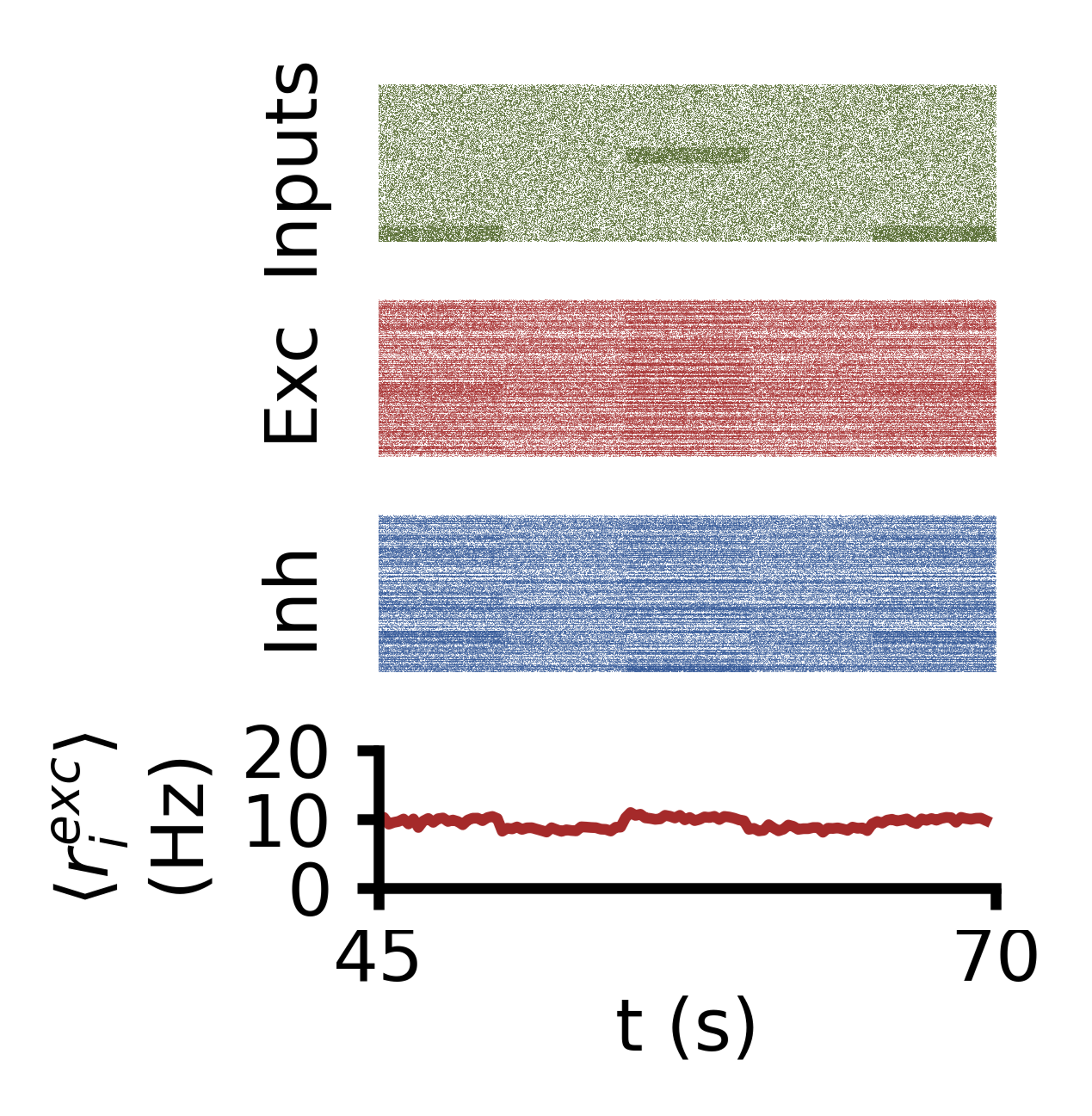

Supplement: S5 Fig — From top to bottom, raster plot of input neurons to a network identical the ones used in Fig 4 and 6, with all connections static; raster plot of excitatory neurons; raster plot of inhibitory neurons; firing rate of the excitatory population (PNG) [file pcbi.1012910.s005.png]

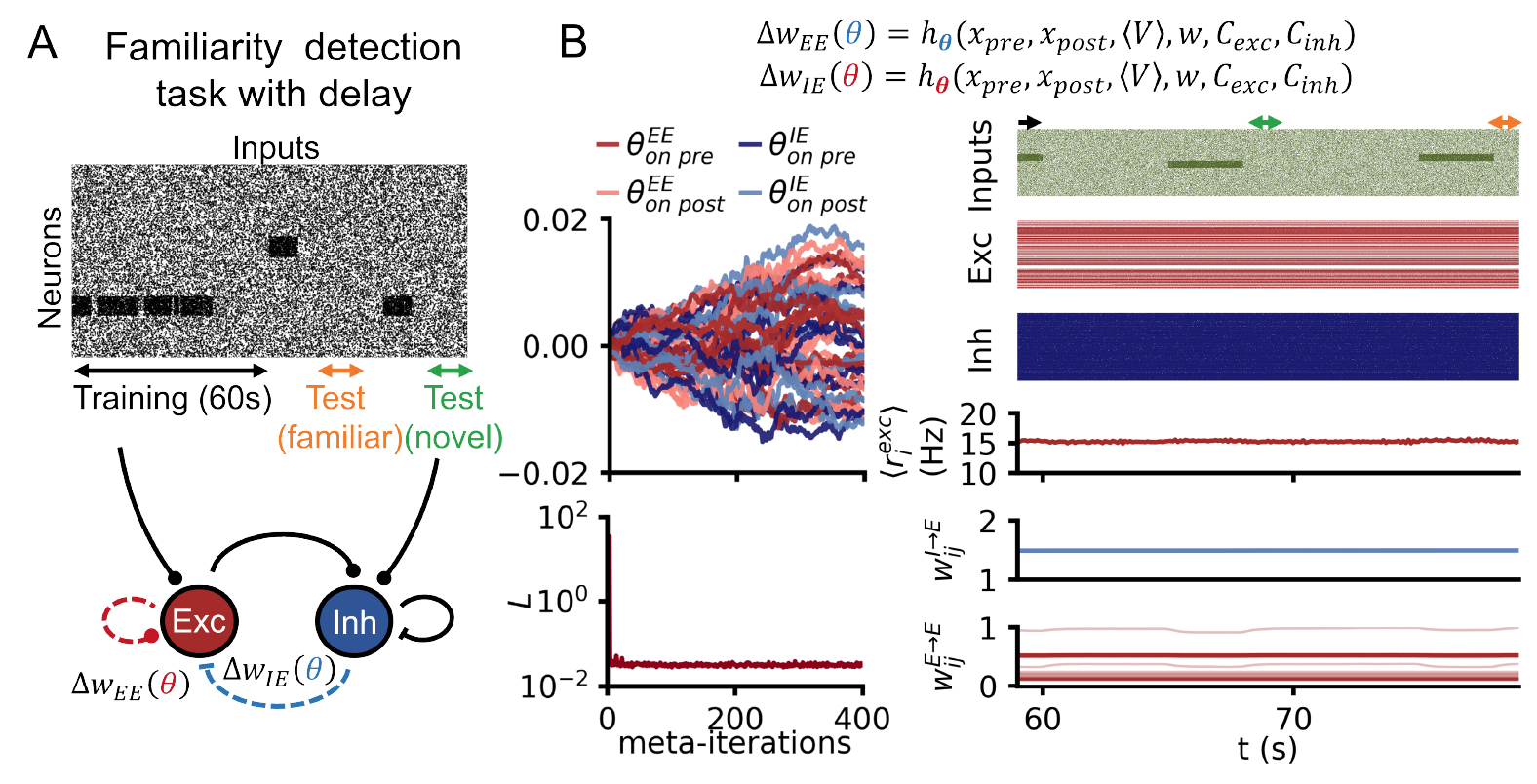

Supplement: S6 Fig — (A) Same network and plasticity search space as in Fig 6, but the task now involves a delay between stimulus presentation and measure of the population activity. (B) Top: evolution of the plasticity parameters across meta-training. The parameters are grouped according to whether they belong to the E-to-E or I-to-E rule, and whether they are part of the weight updates triggered by a presynaptic or by a postsynaptic spike. Bottom: corresponding evolution of the loss function. Right: Network simulated with the learned rule (PNG) [file pcbi.1012910.s006.png]
