# Supplementary material for: Balancing complexity, performance and plausibility to meta learn plasticity rules in recurrent spiking networks
Source: PLoS Comput Biol. 2025 Apr 24;21(4):e1012910. doi: 10.1371/journal.pcbi.1012910 (PMC12021293; doi:10.1371/journal.pcbi.1012910)
Supplement: Attachment — Submitted filename: Response_Letter.pdf [file pcbi.1012910.s007.pdf]

Dear Reviewers,

We thank you for your time and thoughtful comments on our manuscript. We believe we have updated the manuscript accordingly. Below, we discuss the points the reviewers mentioned, grouped by their topics, and sorted in the order of their importance:

1/ “It’s more than just the enormous parameter space that must be "trawled to elicit functions" when applying STDP rules to recurrent SNNs. Some of this isn't related to just parameters of the plasticity rules, but multi-stable solutions of the weight matrices” (R1)

We agree that this issue was not discussed clearly in the manuscript. In our method, there are steps to partially control for these other, non plasticity parameters: we evaluate each plasticity rule in several networks, each with random initial mean weights, connectivity distributions and input rates. We then average over these trials to ensure we find rules with consistent network effects on a range of inputs and initial weight connectivities. We agree that this averaging does not test exhaustively for the more fine-grained initial connectivity motifs studied in Ocker and Doiron 2015, and we do not think our method in its current form would be well-suited. Systematically testing connectivity-rule pairs would bloat the parameter space significantly, rendering meta-learning computationally infeasible and brittle as we saw with the larger synaptic plasticity search spaces (Fig.6&7). We have since moved on to more efficient search methods that allow us to incorporate more intricate network architectures, but this work is outside of the scope of the manuscript at hand. We agree with the reviewer that this is an interesting avenue of investigation but we must kindly ask for patience while we continue to work on this. We also added a paragraph in the discussion detailing the issue above.

2/ “The authors should flesh out with specific examples that they discuss in greater detail about the relationship between these discovered plasticity rules to previous STDP-like curves” (R1). “Is it possible to be firmer on some of the conclusions?” (R2)

We now added comparisons to known STDP rules throughout the revised manuscript. However, the last figure of the paper on degeneracy (and similar results in previous work, Ramesh et al. eLife 2023) urges caution when making predictions about the results we present. Indeed, we find rules with radically different shapes for the same desired network function (Fig. 8), suggesting that there are many equally suitable rules in our plasticity search spaces. Ideally, we would like to know all the rules with the desired function within our search space and compare this population of rules to experimental results. However, obtaining this manifold of functional plasticity rules is impractical with our local optimization method, which is why we are still developing a population optimization method as a result of these shortcomings (Confavreux*, Ramesh* et al. NeurIPS 2023). Here, we wanted to highlight the results we have achieved with local optimisation, only.

3/ “Code availability” (R1 R2)

Our intention was always to release the code before publication, we apologize for the delay, we have now added both the code for the meta-learning strategy, as well as code and optimization results to reproduce the analysis and figures, available at https://github.com/VogelsLab/SpikES

4/ “In particular, is it correct to say that failure to optimise the model was generally due to poor convergence of the method? (R2)

This is a key message of this study and we have rephrased our manuscript to hopefully convey the message better: in our hands, our method decayed in performance for high-dimensional plasticity search spaces (co-active simple rules or more complex single rules) and more complex loss functions. The reasons are 1) the known performance drop of CMA-ES in higher dimensions, 2) an exponential increase in compute time with increased dimensionality (more and more rules need to be tested per meta-iteration), and 3) the need to restart optimization from scratch upon defining a new loss function. These three factors rendered hyperparameter optimization and debugging increasingly impractical. We have added these points of clarification to the discussion.

5/ “You also mention that the objective may not constrain the optimisation sufficiently well, but should a successful optimisation not at least reduce the loss in similar ways? I feel this matters in particular for the co-active rules (where several synapse groups, E-E, I-E etc. are fit simultaneously), as I can see that optimising a single population (i.e. I-I) may not reduce loss when the parameters of the other populations are outside a functional regime.” (R2)

We agree that in theory a poorly constrained objective should be reduced quickly by CMA-ES. However, what we observed in practice was more nuanced: poorly constrained complex plasticity search spaces tended to create “unreliable” network regimes (e.g. Fig.6, note the loss spikes during the optimization and the network dynamics of Fig 6.C for instance), which created losses with high variance (an indication of chaotic loss landscape as seen in other meta-learning work Metz, .., Sohl-Dickstein, ICML 2019). Therefore, in our hands, poorly constrained loss functions also resulted in more arduous optimizations. For co-active rules optimizations, we initialized the plasticity parameters such that there were initially no weight changes (thus ensuring each rule was initially harmless to the network) and added a strong regularization to bias rules towards this harmless, static state if possible. In our hands this only partially improved the optimization.

6/ “To address these questions, it seems prudent to postulate that "simple" parameterizations should be sufficient and informative as such rules can be hand-crafted. What is the lowest order/smallest model that can satisfy the stability constraint? As far as I can see (I may have misread), the experiments for network stabilisation only show optimisation for single populations, but no joint optimisation (Fig 6 appears to show familiarity detection, not stabilisation as suggested in line 227). Can such models be fit by jointly optimising all four synapse populations?” (R2)

We believe meta-learning approaches face a compromise: simple search spaces are the easiest to optimize, however their simplicity makes them already amenable to theoretical analysis and often means we will not discover truly novel rules, thus reducing the appeal of this method. This work constitutes one attempt of pushing the borders of plasticity rule complexity, and acknowledging that with classical, local optimization methods such as CMA-ES, these borders are not far from what is analytically tractable. The conclusions of this study motivated us to develop an optimization method more suited to the problem at hand: keeping all successful solutions and refining the meta-objective as we go instead of restarting from scratch. The resulting method, fSBI (Confavreux* Ramesh* et al. NeurIPS 2023), is a direct scientific heir of this work. In that paper we jointly optimize all four synapse populations, initially considering stability criteria (Fig. 2 in that paper). The reviewer is correct that we presented no optimizations for co-active rules with the stability objective in our manuscript; we have updated the corresponding claim in the discussion. We also mention there that the simplest model that can satisfy the stability constraint could be a single parameter, a

combination of the 6 parameters in the small polynomial search space and using insights from mean-field (Vogels, Sprekeler et al. 2011). We have explored such models in previous work (Confavreux et al NeurIPS 2020, Fig. 3).

7/ “I would expect that the degeneracy shown in Fig. 8 will also appear during optimisation of co-active rules for the stabilisation objective.” (R2)

We agree that degeneracy should be present in any of the co-active cases presented in the manuscript, we now have a full description of this degeneracy of the 4-rule-co-active case (Confavreux* Ramesh* et al. NeurIPS 2023). We believe that, in this manuscript, showing that degeneracy emerges already in the simplest case (single small polynomial rule for stability) is good evidence that degeneracy is a ubiquitous outcome of meta-learning methods. We point this out now, also, in the discussion.

8/ “It is interesting that in this context the parameter covariance is not uniform, as this indicates the presence of "important" and perhaps interpretable directions in parameter space. You seem to interpret this non-uniformity as a weak constraint on the optimisation, but can it also be interpreted in terms of robustness/flexibility? (R2)

We agree (we think, though we are not sure we fully understood the question here) that the non-uniformity of the covariance suggests that some parameters are more important than others during the optimization. Specifically, non-Hebbian parameters do a lot of the heavy lifting in many cases. Thank you for the suggestion to point this out in the manuscript more clearly, which we have followed.

9/ Are there tools to address this further, e.g. methods from Bayesian experimental design (note I'm not suggesting such experiments should be done for this paper)?” (R2)

The reviewer’s suggestion are interesting, and our current project following Confavreux* Ramesh* et al. NeurIPS 2023, is actually to refine our predictions using automated experimental design. We agree with the reviewer here, that new work in this direction would be largely out of scope of the present study.

10/ “I would also semantically just call it an ANN rather than an MLP despite. People might think you’re using actual perceptron units (non-smooth sign functions or heavisides) which likely would create additional discontinuities in your plasticity functions. I was confused about this point until I got to the methods.” (R1)

We understand and agree with the confusion. We initially thought to adapt the reviewers' suggestion. Then, to prevent another confusion of mistaking the ANN with the spiking network, we renamed MLP (to ANNs) to feedforward networks (FFNs). Finally, after having changed the terminology throughout the manuscript, we came to think that neither FFNs nor ANNs were better than MLPs, as “MLP” is a fairly established term even for more generalised perceptrons as in our case, and we found ourselves explaining, “yes, FFN is really a generalised MLP framework” to uninitiated test audiences. We thus changed everything back to MLPs and explained the difference as suggested above upon the introduction of the term in both the methods and the results section. We would appreciate input from both the reviewers on this matter, but we hope this will make things as clear as possible.

11/ “I would describe synchronous regular firing patterns (line 185) as somewhat stereotypical to areas like the hippocampus. Doesn’t this just mean that your result might apply to other brain areas? It’s hard for me to judge how synchronized figure 7 is since the spike raster is so small but there seems to be an overly cortical focus to this paper when other areas don't necessarily operate on E/I balance.” (R1)

We agree that our definition of stability is maybe overly cortical focused, and that some of our results may be more appropriate to other brain areas.

12/ “Is there any reason why the authors did not incorporate terms from the loss function into the meta plasticity rule? some of the huge gains in ML have been through devising new loss functions.” (R1)

We agree that this is an interesting idea, but we have found the SBI approach, in which we can explore the landscape of the parameter space and the loss function more extensively, a great alternative that continues to produce interesting results. In the future we may return to this additional search method, though.

13/ “I would expand on the mean-field results in the main section, it reads like too much of a throw away when it provides intuition as to why I-I connections can’t directly control E-firing rates.” (R1)

We respectfully disagree here, because we make only small contributions to an already well-established method for the question at hand, and did not want to create the appearance of taking credit for other people's work. In fact, thanks to the reviewer’s suggestion, we have revisited the manuscript in the respective places to cite the existing body of work more extensively, and fleshed out the relevant mean-field method section of the manuscript.
